# Homeopathic medical practice for anxiety and depression in primary care: the EPI3 cohort study

**DOI:** 10.1186/s12906-016-1104-2

**Published:** 2016-05-04

**Authors:** Lamiae Grimaldi-Bensouda, Lucien Abenhaim, Jacques Massol, Didier Guillemot, Bernard Avouac, Gerard Duru, France Lert, Anne-Marie Magnier, Michel Rossignol, Frederic Rouillon, Bernard Begaud, for the EPI3-LA-SER group

**Affiliations:** LA-SER, 10 place de Catalogne, 75014 Paris, France; Conservatoire national des arts et métiers (CNAM), 292 Rue Saint-Martin, 75003 Paris, France; LA-SER Europe Limited, 66 Chiltern St, London, W1U 4JT UK; Department of Epidemiology, London School of Hygiene & Tropical Medicine, Keppel St, Bloomsbury London, WC1E 7HT UK; Faculty of Medicine, University of Franche Comté, 9 Rue Ambroise Paré, 25000 Besançon, France; Pasteur Institute, 28 rue du Docteur Roux, 75015 Paris, France; University of Paris-île-de-France-Ouest, 9 boulevard d’Alembert 78280, Guyancourt, France; Cyklad group, 16 Rue André Le Nôtre, 69140 Rillieux-la-Pape, France; INSERM U1018, Centre for Epidemiology and Population Health, Hôpital Paul Brousse, Bât 15/16, 16 Avenue Paul Vaillant Couturier, 94807 Villejuif, France; Faculty of Medicine, University Pierre and Marie Curie, 4 Place Jussieu, 75005 Paris, France; Department of Epidemiology, Biostatistics and Occupational Health, McGill University, Purvis Hall, 1020 Pine Avenue West, Montreal, QC H3A 1A2 Canada; Sainte-Anne Hospital, University of Paris V René Descartes, 100 rue de la Santé, 75674 Paris, France; INSERM U657, University Victor Segalen Bordeaux 2, 146 rue Léo Saignat, 33076 Bordeaux, France

**Keywords:** Anxiety, Depression, Homeopathy, Primary care

## Abstract

**Background:**

The purpose of the study was to compare utilization of conventional psychotropic drugs among patients seeking care for anxiety and depression disorders (ADDs) from general practitioners (GPs) who strictly prescribe conventional medicines (GP-CM), regularly prescribe homeopathy in a mixed practice (GP-Mx), or are certified homeopathic GPs (GP-Ho).

**Methods:**

This was one of three epidemiological cohort studies (EPI3) on general practice in France, which included GPs and their patients consulting for ADDs (scoring 9 or more in the Hospital Anxiety and Depression Scale, HADS). Information on all medication utilization was obtained by a standardised telephone interview at inclusion, 1, 3 and 12 months.

**Results:**

Of 1562 eligible patients consulting for ADDs, 710 (45.5 %) agreed to participate. Adjusted multivariate analyses showed that GP-Ho and GP-Mx patients were less likely to use psychotropic drugs over 12 months, with Odds ratio (OR) = 0.29; 95 % confidence interval (CI): 0.19 to 0.44, and OR = 0.62; 95 % CI: 0.41 to 0.94 respectively, compared to GP-CM patients. The rate of clinical improvement (HADS <9) was marginally superior for the GP-Ho group as compared to the GP-CM group (OR = 1.70; 95 % CI: 1.00 to 2.87), but not for the GP-Mx group (OR = 1.49; 95 % CI: 0.89 to 2.50).

**Conclusions:**

Patients with ADD, who chose to consult GPs prescribing homeopathy reported less use of psychotropic drugs, and were marginally more likely to experience clinical improvement, than patients managed with conventional care. Results may reflect differences in physicians’ management and patients’ preferences as well as statistical regression to the mean.

## Background

Anxiety and depressive disorders (ADDs) are highly prevalent worldwide and represent a leading reason for consultation in primary care [1, 2]. Although systematic reviews and guidelines [3, 4] recognise the efficacy of antidepressant and psychotropic drugs for specific ADDs, heterogenity in the diagnostic approach of these patients in primary care is partly responsible for the non-optimal utilization of these drugs, particularly in mild and moderate cases [5]. The prevalence of ADDs in homeopathic care also ranks high, only surpassed by low-back pain [6]. Patients who seek homeopathic care differ from those preferring conventional medicine, but the diagnostic make-up of their consultations has been described as similar [7]. Evidence summarized in systematic reviews conducted to assess the benefit of homeopathy in ADDs is too limited to sufficiently draw firm conclusions regarding the efficacy or effectiveness of homeopathy in this indication [8–11]. However, its potential to reduce psychotropic drug utilization in ADD patients who opt for this type of care has been identified with minimal adverse effects, and potential economic impacts on healthcare resources [12, 13]. In France, homeopathy is practiced exclusively by physicians and is partly reimbursed by National Health Insurance. Physicians who prescribe homeopathy are either occasional prescribers or have completed a certification in homeopathic medicine accessible through private organisations (see, eg, www.cedh.org/).

This 1-year, population-based cohort study compared patients’s real-life utilization of conventional psychotropic drugs and clinical progression of ADDs between those seeking care from general practitioners (GPs) who rarely prescribe homeopathy (strict prescribers of conventional medicines) (GP-CM), and those seeing regular prescribers of homeopathy within a mixed practice (GP-Mx) or certified homeopathic GPs (GP-Ho).

## Methods

### Study design and selection of study subjects

This cohort study was conducted in France between 2007 and 2008 within the nationwide EPI3 survey of primary care practice in a representative sample of GPs and their patients [1]. The EPI3 survey included three epidemiological follow-up cohort studies (hence the name EPIdemiologic study – three cohorts) of common reasons for consultation in primary care, one of which focused on patients with ADDs (the two others being musculoskeletal disorders and respiratory infections). The EPI3 survey was an observational study where no instructions were given to participating physicians or patients in order to prevent interference in usual clinical practice. The sample was drawn using a two-stage sampling process. First, a random sample of GPs was drawn from the French National Directory of Physicians in primary care. Sampling of GPs was stratified according to their declaration of prescribing preferences, obtained by telephone at the time of recruitment and categorized into three groups: strict prescribers of conventional medicine (GP-CM) who declared they never or rarely used homeopathy; regular prescribers of homeopathy in a mixed practice (GP-Mx); and certified homeopathic GPs (GP-Ho). This classification of GPs by type of management served as the basis for comparing their patients. As GPs in the three groups were free to prescribe conventional and/or homeopathic drugs, this study did not compare patients by the type of prescription issued but only by the type of physician (prescribing preferences) they have chosen to consult. The second-stage sampling consisted of a 1-day survey of all patients attending the medical practice of each participating GP during which a trained research assistant surveyed all patients in the waiting room. For this cohort study, consenting adult patients consulting for all types of anxiety and depression symptoms were invited to a baseline telephone interview within 72 h of recruitment, which included the French adaptation of the Hospital Anxiety and Depression Scale (HADS) questionnaire [14, 15]. The HADS is composed of two sub-scales, one for anxiety and one for depression. The subscales were only used for describing the study population. All other analyses used the combined HADS anxiety and depression scores. Patients scoring nine or more in the HADS questionnaire were then invited to follow-up interviews at 1, 3 and 12 months. Diagnoses of anxiety and depression symptoms by GPs were based on the GPs’ own clinical judgement, with no attempt at standardization or external validation. This strategy was aimed at being representative of all patients consulting for ADD symptoms who were the most likely to receive a prescription for a psychotropic drug in real-life primary care.

### Information collected

At inclusion, GPs completed a medical questionnaire for each patient surveyed, including the main reason for consultation and up to five other diagnoses (co-morbidities), and all drugs prescribed that day. Diagnoses were coded by a trained archivist using the ninth revision of the International Classification of Diseases. All consenting patients completed a self-administered questionnaire at inclusion (waiting room), collecting information on lifestyle, occupation, hospitalization history, number of GP consultations in the past year, and the health-related quality of life Short Form-12 (SF-12) [16]. Follow-up telephone interviews included the HADS and spanned the patient’s history since the previous interview regarding drug utilization (conventional and homeopathy) and injuries (resulting from a fall, motor vehicle collision, sport, or occupation). Moreover, the 12-month questionnaire evaluated lifetime history of suicide attempts, specifying any such occurrence since entry into the cohort. Drug utilization, whether prescribed or obtained over the counter or from the family pharmacy, was assessed using a standardized method known as Progressive Assisted Backward Active Recall (PABAR), previously validated against medical prescriptions [17, 18], and drugs were automatically recorded using the anatomical therapeutic chemical classification index (ATC), 2009 revision. For patients participating in this ADD cohort study, particular emphasis was put on psychotropic and homeopathic drugs commonly used in ADDs by specifically asking patients if they had taken any drug from a list of 36 products that was read to them after they had spontaneously reported medication taken in that period.

### Statistical analysis

Differences at baseline between GP-CM, GP-Mx and GP-Ho groups were assessed using multivariate logistic regression analyses. A propensity score was computed for each participant in the study on their probability of belonging to either GP-Mx or GP-Ho groups compared to the GP-CM group, according to all variables listed in Table 1. The score was used to adjust for differences between the groups in all subsequent analyses. Given the imbalance between the groups for severity of ADD at baseline, analyses were stratified in two groups using the HADS score from 9 to 11 and 12 and above [19].

**Table 1:** Baseline characteristics of patients with anxiety and depressive disorders (ADDs) by type of medical practice^a^ (*N* = 710)

	Total %	GP-CM %	GP-Mx %	GP-Ho %	Non-participating patients %
	(*N* = 710)	(*N* = 161)	(*N* = 260)	(*N* = 289)	(*N* = 852)
Female gender	79.7	73.9	78.9	**83.7** ^**^	**72.6** ^*^
Age (years)					
18–39	23.5	23.6	23.1	23.9	24.9
40–59	47.3	42.9	48.9	48.4	43.4
60+	29.2	33.5	28.1	27.7	31.7
Body Mass Index (kg/m^2^)					
<25	59.7	52.2	53.5	**69.6** ^**^	58.9
25–29	26.9	30.4	31.1	**21.1**	27.6
30+	13.4	17.4	15.4	**9.3**	13.5
Smoking					
Never smoked	51.6	50.3	51.2	**52.6** ^**^	49.1
Former smoker	23.6	23.0	22.6	**24.9**	24.0
Smoker	24.8	26.7	26.2	**22.5**	26.9
Alcohol consumption					
Rarely/never	35.6	39.8	37.3	31.8	33.7
Once a week	52.1	48.5	51.9	54.3	53.0
Daily	12.3	11.7	10.8	13.9	13.3
Physical activity					
30 min and more	26.8	27.3	25.8	27.3	30.5
Education					
Beyond secondary school	48.3	37.9	43.1	**58.1** ^**^	46.7
Occupational status					
Employed	51.1	42.9	51.9	55.0	51.4
Unemployed	15.2	14.9	17.3	13.5	12.8
Retired	33.7	42.2	30.8	31.5	35.8
Complementary health insurance (CMU)	6.62	11.2	5.4	5.2	8.80
GP declared as the regular treating physician	67.8	84.5	83.1	**47.1** ^**^	73.0

*GP* general practitioner

^a^Type of medical practice according to physicians’ prescribing preferences: *GP-CM* conventional medicine; *GP-Mx* mixed, conventional and homeopathic practice; *GP-Ho* registered homeopathic physicians

^*^Difference statistically significant (*P* <0.05) in multivariate logistic regression comparing participants to non-participants

^**^Differences with the group GP-CM (reference) statistically significant (*P* <0.05) in multivariate logistic regression in a saturated model including all variables in the table

At each follow-up, a patient was declared to have clinically improved if the HADS score fell below the value of nine. Consumption of psychotropic drugs was defined at each interview interval as the proportion of patients declaring at least one utilization, since the previous interview, of drugs belonging to the ATC classes N05B (anxiolytics), N05C (hypnotics and sedatives) and N06A (antidepressants). ADD’s clinical improvement at the 12-month follow-up, utilization of psychotropics and occurrence of traumatic events (any during the 12-month follow-up) were compared across the three groups using the GP-CM group as the reference in logistic regression adjusted for baseline characteristics (propensity score) and stratified for the severity of the ADD at baseline. Clustering effects resulting from recruiting several patients consulting the same GP, and autocorrelation between responses to the four consecutive interviews, were controlled for using Generalized Estimating Equations (GEE) in the multivariate models. The Odds ratios produced (and their 95 % confidence interval (CI)) are then interpreted as the differences in the likelihood of clinical improvement and use of psychotropic drugs in the groups GP-Mx and GP-H0 compared to the group GP-CM. All analyses were performed using SAS version 9.1 (SAS Institute, Inc., Cary, North Carolina, USA).

## Results

### Study population

The general EPI3 health survey included 825 GPs and 8559 patients. Among the patients, 1562 adults fulfilled the specific inclusion criteria established for the ADD cohort and 710 (45.5 %) who scored nine or higher on the HADS agreed to participate and responded to at least one follow-up interview, of which 660 were diagnosed by the physician with an anxiety disorder and 467 with a depressive disorder (not mutually exclusive). Participants were slightly more often females (79.7 %) than were non-participants (72.6 %), but the two groups were very similar otherwise (Table 1). Compared to the GP-CM group, patients who consulted a GP-Ho were more often non-smoking females with a higher level of education and a lower body mass index (BMI); differences that were statistically significant after taking into account all other factors (Table 1). In addition, GP-Ho participants were less likely to report the attending GP as their regular physician (47.1 %) compared to GP-CM (84.5 %). Overall, no significant difference was observed between patients of the GP-Mx and GP-CM groups.

### Clinical characteristics at baseline

Compared to the patients in the GP-CM group, those consulting a GP-Ho were less likely to have a HADS score equal to or above 12 (52.3 % versus 57.8 %, respectively), to have a history of suicide attempts (14.2 % versus 23.0 %, respectively), another ADD (53.6 % versus 60.3 %, respectively) or primary insomnia (34.3 % versus 40.4 %, respectively), all differences being statistically significant (Table 2). Patients in the GP-Ho group also had a lower number of co-morbidities, and fewer visits to a GP and hospitalizations in the previous year, compared to the GP-CM group. The profile of GP-Mx patients was similar to that of GP-CM patients, except for history of suicide attempt and hospitalization (all causes) and a concomitant ADD, for which they resembled patients from the GP-Ho group. Prescribing preferences of physicians in the three groups were confirmed at baseline by their respective prescribing rates of conventional psychotropic drugs (GP-CM: 80.8 %; GP-Mx: 71.9 %; GP-Ho: 32.9 %) and homeopathic drugs (GP-CM: 0.0 %, GP-Mx: 13.1 % and GP-Ho: 55.7 %).

**Table 2:** Baseline medical characteristics of patients with anxiety and depressive disorders (ADDs) by type of medical practice^a^ (*N* = 710)

	GP-CM %	GP-Mx %	GP-Ho %
	(*N* = 161)	(*N* = 260)	(*N* = 289)
Patients with anxiety disorders	90.7	92.7	94.5
Patients with depressive disorders	69.6	70.8	59.2
HADS score [mean (SD)]			
Anxiety score	11.9 (2.2)	12.0 (2.2)	11.8 (2.2)
Depression score	11.2 (2.0)	11.0 (1.8)	10.8 (1.8)
Severe ADD at inclusion (HADS ≥12)			
All patients	57.8	55.4	**52.3** ^*^
Patients with anxiety disorder	47.8	48.1	45.7
Patients with depressive disorder	26.7	21.9	**17.0** ^*^
History of suicide attempts (lifetime)	23.0	**12.4** ^*^	**14.2** ^*^
Concomitant ADD (other than main diagnosis)	60.3	63.5	**53.6** ^*^
Primary insomnia	40.4	**27.3** ^*^	**34.3** ^*^
Physical co-morbidities			
Cardiovascular or metabolic disorders	35.4	32.7	**22.2** ^*^
Respiratory diseases	9.9	5.8	7.3
Musculoskeletal disorders	19.9	19.6	22.5
Diabetes and other endocrine disorders	14.3	11.5	**6.6** ^*^
Digestive disorders	13.0	8.1	11.8
History of hospitalisation	29.8	**20.0** ^*^	**20.1** ^*^
Number of visits to the GP in the previous year			
0–6	55.3	49.2	**65.7** ^*^
7+	44.7	50.8	**34.3**
Prescription of psychotropic drug at inclusion visit	80.8	**71.9** ^*^	**32.9** ^*^
Prescription of homeopathy^b^ at inclusion visit	0	**13.1** ^*^	**55.7** ^*^
Quality of life (SF-12)			
Mental score (mean, SD) [mean (SD)]	33.0 (8.7)	33.5 (9.1)	34.3 (9.2)
Physical score (mean, SD)	45.2 (9.4)	46.7 (10.0)	49.0 (8.7)

*GP* general practitioner, *HADS* Hospital Anxiety and Depression Scale, *SD* standard deviation

^a^Type of medical practice according to physicians’ prescribing preferences: GP-CM, conventional medicine; GP-Mx, mixed, conventional and homeopathic practice; GP-Ho, registered homeopathic physicians

^b^Homeopathic preparation specific for ADD

^*^Differences statistically significant (*P* <0.05) with the group GP-CM (reference) in multivariate logistic regression including propensity score and all variables in Table 2

### Clinical evolution and outcomes

**Fig. 1:**
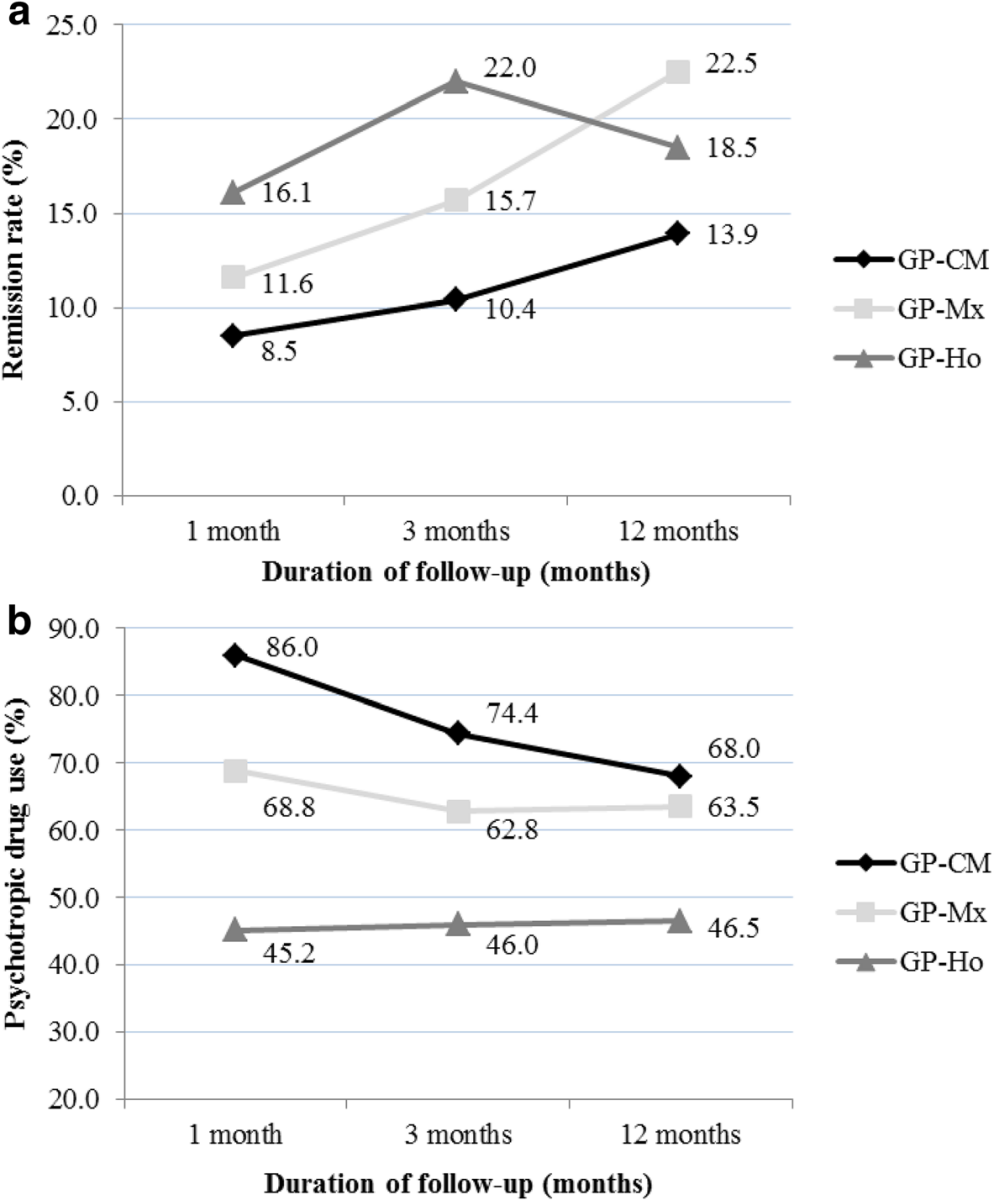
Proportions of patients experiencing clinical improvement^§^ of their anxiety and depressive disorders (**a**) and proportions of those who used at least one psychotropic drug (**b**) during the 12-month follow-up by type of medical practice* (*N* = 710). *Type of medical practice according to physicians’ prescribing preferences: *GP-CM* conventional medicine (reference group); *GP-Mx* mixed, conventional and homeopathic practice; *GP-Ho* registered homeopathic physicians. ^§^Clinical improvement defined as a HADS score falling below the value of 9

Figure 1 shows, for each group of patients, the evolution of crude proportions for ADD clinical improvement (HADS <9) at each follow-up point (Fig. 1a), and proportions of patients reporting at least one psychotropic drug utilization (Fig. 1b). Trends towards clinical improvement over time were statistically significant within each group (Chi-square for trend: *P* <0.001), with faster clinical evolution for the GP-Ho group in the first 3 months, and highest improvement rate for the GP-Mx group at 12 months. Crude clinical improvement rates remained the lowest in the GP-CM group throughout follow-up. Crude trends in psychotropic drug utilization in the GP-CM and GP-Mx groups shadowed clinical evolution to reach relatively similar values at 12 months (68.0 and 63.5 % respectively), while it remained well under 50 % at all times in the GP-Ho group.

After controlling for potential confounders and baseline characteristics, the probability of ADD clinical improvement (HADS <9) over the 12-month follow-up was 1.7 times more likely among GP-Ho patients compared to GP-CM [OR = 1.70 (95 % CI: 1.00 to 2.87)], a result of borderline statistical significance (Table 3). The probability of clinical improvement among GP-Mx patients did not differ from that observed in GP-CM patients after controlling for all other factors [OR = 1.49 (95 % CI: 0.89 to 2.50]. Consumption of psychotropic drugs was significantly lower in both GP-Ho and GP-Mx groups compared to the GP-CM group, being respectively less than one third as likely [OR = 0.29 (95 % CI: 0.19 to 0.44)] and less than two thirds as likely [OR = 0.62 (95 % CI: 0.41 to 0.94)]. These effect sizes were not affected by the severity of ADD at baseline, with similar results for patients with initial HADS scores above and below the value of 12 within each group. Self-reported injuries and suicide attempts were insufficient in number to allow modelling in regression analyses; 14.8, 7.11 and 9.45 % declared injuries while 4.97, 1.92 and 1.38 % declared a suicide attempt in the GP-CM, GP-Mx and GP-Ho groups, respectively. None of those differences was statistically significant in unadjusted statistical analyses.

**Table 3:** Twelve-month clinical progression and psychotropic drug utilisation for patients with anxiety and depressive disorders by type of medical practice^a^ (*N* = 710)

	GP-Mx vs GP-CM	GP-Ho vs GP-CM
	OR^c^ [95 % CI]	OR^c^ [95 % CI]
	(*p* value)	(*p* value)
Clinical improvement (HADS score <9^b^)		
All patients^d^	1.49 [0.89–2.50]	1.70 [1.00–2.87]
	(0.13)	(0.05)
HADS ≥12 at inclusion	1.52 [0.69–3.34]	1.70 [0.81–3.55]
	(0.30)	(0.16)
HADS <12 at inclusion	1.34 [0.66–2.72]	1.61 [0.77–3.38]
	(0.42)	(0.21)
Psychotropic drug use^b^		
All patients^d^	0.62 [0.41–0.94]	0.29 [0.19–0.44]
	(0.02)	(<0.001)
HADS ≥12 at inclusion	0.63 [0.38–1.07]	0.29 [0.18–0.48]
	(0.08)	(<0.001)
HADS <12 at inclusion	0.65 [0.32–1.31]	0.33 [0.15–0.66]
	(0.23)	(0.003)

^a^Type of medical practice according to physicians’ prescribing preferences: *GP-CM* conventional medicine (group of reference); *GP-Mx* mixed, conventional and homeopathic practice; *GP-Ho* registered homeopathic physicians

^b^ HADS score less than 9 at the 12-month follow-up; any psychotropic drug use declared in the 12-month follow-up

^c^Odds ratios and 95 % confidence intervals obtained from GEE models adjusted for propensity score including all variables in Tables 1 and 2

^d^Odds ratios for all patients may not lie between those obtained for the two severity subgroups as they result from separate multivariate analyses and were shown to have wide confidence intervals

## Discussion

This population-based, prospective cohort study described the real-life clinical management of patients with ADD consulting primary care physicians with prescribing preferences for homeopathy. Our results first showed that patients who chose GP-Ho differed substantially from other patients, a finding well described elsewhere [20]. The original finding of this large cohort study is that the lower use of conventional psychotropic drugs in that group of patients did not compromise the evolution of their ADD once stratified by its severity at baseline. Indeed, patients in the GP-Ho group were marginally more likely to experience clinical improvement than patients managed with conventional care. Beside the different nature of these patients, two other factors could explain the results. First, homeopathic physicians probably diagnose ADDs differently and may transmit different information to their patients regarding prognosis. In the context of the present study, homeopathy is practiced by GPs who are all trained in conventional medicine and have access to all diagnostic and therapeutic resources recommended for ADDs in primary care. The GP-Ho group of physicians represented a distinct type of management and patient–physician interaction, which in itself may account for a large part of the therapeutic results observed. In addition, inefficacy of psychotropic drugs and statistical regression to the mean are two other potential contributions to the results.

A second explanation derives from the acknowledgment that conventional drugs lack efficacy in a large number of ADD patients seen in primary care [21]. In addition, although clinical guidance on ADD is widely available in general practice [4, 22], there is evidence to suggest a lack of implementation in real-life situations with regard to access to treatment and to poor patient compliance with psychotropic drugs, including early drop-out from treatment and overdosing [5, 23–25]. Therefore, a lower utilization of a largely ineffective therapy should not compromise the clinical evolution of ADD overall. In those conditions, homeopathy can be viewed as an additional tool to support patients inclined to use this type of care without resorting to conventional drugs when they are not indicated. The potential positive impact on healthcare costs of this drug-sparing strategy remains to be estimated at the population level [12, 13].

Another important difference for patients in the GP-Ho group was the low proportion (47.1 %) declaring the attending physician as their regular GP, nearly half of that observed in the GP-CM and GP-Mx groups (84.5 and 83.1 %, respectively). One potential explanation for this difference might be that some patients who consult in homeopathic care do so after a subjective perception of failure to improve with the treatment prescribed by their regular physician, an effect that has been observed elsewhere [26], or who do not see a GP regularly. However, the influence of healthcare prior to entry in this cohort study, positive or negative, could not be assessed and remains only a potential explanation for the results.

Systematic reviews have shown inconsistent results for the effectiveness of homeopathy in depression and anxiety. The quality of the evidence is limited in part by inadequate study designs and insufficient number of patients [8, 9]. The results of this study cannot be interpreted as effectiveness of conventional against homeopathic care. Patients in the three groups of physicians were compared on the basis of the prescribing preferences for homeopathy by their GPs, all of whom were free to prescribe conventional drugs also (indeed, 32.9 % of GPs certified in homeopathy did prescribe a conventional psychotropic drug at inclusion). The category of GPs who declared themselves as regular prescribers of homeopathy without being trained as homeopathic practitioners (GP-Mx) became an interesting group as it allowed the observation of patients in a naturally mixed setting combining homeopathic and conventional medicine. That group differed little from the GP-CM group, which would indicate that it was mostly the type of management rather than the type of prescription that might explain the results. Patients in the GP-Mx group also used less psychotropic drugs, indicating that patients’ preference for homeopathy plays an important role in these results, as suggested by some authors [27, 28].

### Strengths and limitations

As the study was appended to a population health survey, it provided a unique opportunity to assemble a pool of potential patients seen in primary care, with no selection criteria applied prior to the invitation to join this cohort study. The distribution of physicians and patients participating to the EPI3 general survey was close to what is known about medical demography and reasons for consultation in France [1]. A strength of this study was the variety of information collected at baseline, covering socio-demographic and clinical characteristics which allowed the differences between the groups to be described in detail. Care was also taken to ensure quality of outcome measures. HADS has been shown to be highly sensitive and specific for diagnoses of ADDs in previous primary care studies [15]. Drug utilization was obtained from patient interviews using a methodology that had been previously validated, although not specifically for psychotropic drugs [17, 18]. The quality of psychotropic drug reporting should not differ much across the three groups of patients, as they were unaware of the specific hypotheses regarding drug consumption. The methodology has shown excellent recall capacity up to 2-year follow-up and has the advantage of identifying drugs purchased over the counter and from the family pharmacy, which are not accounted for in prescription databases and potentially represent an important source of psychotropic drug utilization [29]. It should be noted that while psychotropic drug utilization described at baseline (Table 2) was obtained from physicians’ reports, patients’ reporting was preferred for the analyses of evolution of psychotropic utilization over time.

One of the main limitations of this study was its low participation rate, at 45 % of eligible patients. Although generally considered acceptable for a general health survey of this type, in which patients are asked to participate in a 1-year follow-up study, the participation rate leaves the results open to potential selection bias. Differences between participants and non-participants were small, however, and participation rates were almost identical across the three groups of GPs, which made comparison biases unlikely. Also, co-morbidity and SF-12 scores were similar between the groups and consistent with other French and European studies [30–33]. Nevertheless, sample size was sufficient to compare groups for psychotropic drug utilization and rates of clinical improvement, but not for safety outcomes such as occurrence of injuries and suicide attempts that were too few to allow proper statistical modelling.

Another limitation of this study was the nature of patients’ ADD symptoms and GPs’s diagnoses; these were the basis for patients’ recruitment and could have differed in the three groups of physicians. The recruitment strategy was chosen to reflect primary care practice in real life, but could have introduced a bias with systematic differences in the diagnostic make-ups of the groups. In effect, patients in the GP-Ho group had less severe ADD on almost all indicators, including the lifetime history of a suicide attempt, which was almost half in the GP-Ho and GP-Mx groups compared to the conventional practice (GP-CM) group (Table 2). This imbalance at baseline could explain a lower use of psychotropic drugs (bias by indication) and better clinical outcomes in those two groups, even when controlling for this potential bias in the analyses. Stratification of the analyses in lower and higher HADS patients at inclusion was an attempt to better view the effect of this potential bias. Similarity of results in the two populations of patients indicated that baseline differences alone could probably not explain the results.

## Conclusions

Patients with ADDs choosing to consult GPs who prescribe homeopathy in addition to conventional medicine reported use of fewer psychotropic drugs and were marginally more likely to experience clinical improvement than patients managed with conventional care. These findings may result from the combined effect of inefficacy of conventional psychotropic drugs and statistical regression to the mean as well as from effective homeopathic management.

## Declarations

### Ethics approval and consent to participate

The study was approved by the French National Data-Protection Commission (CNIL) and the French National Medical Council (CNOM). Participating physicians received compensation fees for their participation. Patients were not compensated for their participation in the study. All patients have given informed consent to take part in the study.

### Consent for publication

Not applicable.

### Availability of data and materials

None available.

## Abbreviations

AAD: anxiety and depression disorder
ATC: anatomical therapeutic chemical
BMI: body mass index
CI: confidence interval
CMU: complementary health insurance
CNIL: French National Data-Protection Commission
CNOM: French National Medical Council
EPI3: EPIdemiologic study – 3 cohorts
GEE: generalized estimating equations
GP: general practitioner
GP-CM: general practitioner who strictly prescribes conventional medicines
GP-Ho: certified homeopathic GP
GP-Mx: general practitioner who regularly prescribes homeopathy in a mixed (conventional and homeopathic) practice
HADS: Hospital Anxiety and Depression Scale
OR: odds ratio
PABAR: progressive assisted backward active recall
SF-12: short form-12
